# Anxiety Levels among Polish and Turkish Dentists during the COVID-19 Pandemic

**DOI:** 10.3390/healthcare10020357

**Published:** 2022-02-11

**Authors:** Iwona Olszewska-Czyz, Sarkis Sozkes

**Affiliations:** 1 Periodontology, Prophylaxis and Clinical Oral Pathology Department, Jagiellonian University Medical College, 31155 Krakow, Poland; 2 Biomaterials Department, Biomedical Engineering Corlu Faculty, Tekirdag Namik Kemal University, Tekirdag 59860, Turkey; ssozkes@nku.edu.tr

**Keywords:** COVID-19 pandemic, anxiety, dentist

## Abstract

Working conditions, work-related stressors and high risk of infection, as well as the fear of contagion and spreading the disease to family members, may have influenced dentists’ mental health during the COVID-19 pandemic. The aim of this study was to evaluate the anxiety levels among Polish and Turkish dentists during the COVID-19 pandemic and to investigate any relevant predictors. The study was an anonymous online questionnaire-based cross-sectional study that was conducted among dentists from two countries: Poland and Turkey. In total 400 dentists (200 from each country) participated in the study. The survey consisted of two parts: part 1 comprised demographic data, including age, gender, country of origin, COVID-19 infection history, place of work and lockdown history; part 2 was based on the State–Trait Anxiety Inventory (STAI). The mean trait and state anxiety levels of the Polish dentists was statistically significantly lower than that of the Turkish dentists (*p* = 0.000; *p* < 0.05). However, Polish dentists had higher state anxiety levels than trait levels, while both types of anxiety among Turkish dentists were almost at the same level. The number of dentists who suffered from COVID-19 was found to be statistically significantly higher in Poland (54%) than in Turkey (16%) (*p* = 0.000; *p* < 0.05). The percentage of dentists reporting anxiety was 51% in Poland and 95.5% in Turkey. Polish dentists reported a lower mean anxiety level during the COVID-19 pandemic than the dentists in Turkey but their anxiety levels were more affected by the COVID-19 pandemic as they had a higher difference between their state and trait anxiety levels. The higher coronavirus infection rate and lack of governmental lockdowns of dental practices during the pandemic in Poland compared with those in Turkey may explain the difference in the state and trait anxiety levels.

## 1. Introduction

### 1.1. Background

Since the World Health Organization (WHO) declared the spread of coronavirus disease 2019 (COVID-19) a pandemic, healthcare institutions have prioritized critical patient care in order to reduce viral transmission and testing [1]. Meanwhile, lockdowns, isolation, fear of disease, economic recession and job loss were also negatively affecting the mental health of many people [2,3]. A high number of individuals reported that stress over COVID-19 led to specific negative impacts on their wellbeing, such as the difficulty in sleeping or eating and worsening of chronic conditions. Numerous studies have also shown that among the COVID-19 mental health consequences, anxiety and depressive disorders played a crucial role [4,5,6,7,8,9,10,11,12].

Anxiety is a normal reaction to uncertainty and in the short term prepares an individual to face an intense situation by increasing breathing and heart rate, as well as blood flow to the brain. However, an excessive or persistent state of anxiety can have a devastating effect on physical and mental health. Symptoms of an anxiety disorder may begin immediately or years later. Long-term anxiety is harmful for mental and physical health and leads to the release of stress hormones on a regular basis, which can increase the frequency of symptoms such as headaches, dizziness and also depression [2,3,4,5].

Due to the nature of their work, healthcare workers (HCWs) face not only a high occupational risk of infection but also negative impacts on their mental health due to the COVID-19 pandemic. The influence of previous pandemics on the mental health of healthcare providers has been well documented [13,14,15,16,17,18,19]. Studies from the 2003 SARS pandemic reported higher levels of depression [16], anxiety [17], post-traumatic stress disorder [18], burnout [19] and stress [3] among HCWs, with symptoms persisting up to 1 year after the pandemic ended [3,19].

Dental professionals are an important part of the healthcare system due to the oral–systemic link. Many diseases and medications impact oral health and vice versa pathologic conditions in the mouth influence systemic wellbeing [20,21,22,23,24]. The specific working conditions of dental professionals present a high risk of infection. Dentists are directly exposed to pathogenic microorganisms, including viruses and bacteria, that infect not only the oral cavity but also the respiratory tract. The dental care environment, which involves face-to-face communication with patients and exposure to saliva, blood, other body fluids and airborne microorganisms suspended in the air for long periods, as well as handling sharp instruments, carries the risk of contagion [20]. Moreover, working conditions, work-related stressors and the high risk of infection, as well as the fear of contagion and spread to family members, may have influenced dentists’ mental health during the COVID-19 pandemic [25,26,27,28,29,30,31,32,33,34].

There are many differences between Poland and Turkey in several aspects, starting from the demographic and ending up in the COVID-19 dynamics. There are also some differences in their dental care systems. For example, there is a similar number of dentists in both countries but Turkey’s population is twice the size of Poland’s [35]; there are over 38,000 dentists in Poland [36] and nearly 40,000 dentists in Turkey [37]. As for COVID-19 history, at the time of writing Poland had a total of 2,881,046 SARS-CoV-2 infection cases with 75,179 deaths [38] while Turkey reported a total number of 5,493,244 cases with 50,324 deaths overall [39]. However, Poland and Turkey were at a different stage in the COVID-19 pandemic at the moment of conducting the study; hence, the COVID-19-related dynamics would have differed between the two countries. There were less than 100 cases of confirmed COVID-19 infections with only a few COVID-19-related deaths per day at the time of writing in Poland, while Turkey reported over 6000 cases of confirmed COVID-19 infections with 46 COVID-19-related deaths per day [38,39] for the same time period. As for the COVID-19 vaccination rate, at the time the research took place it was reported that 45.2% of the adult population had received at least one dose of a COVID-19 vaccine in Poland compared to 46.5% in Turkey [38,39].

Considering all above aspects, a hypothesis of the relationship between the COVID-19 pandemic and anxiety levels of dentists has been raised, running parallel to possible differences between countries and other factors.

### 1.2. Objectives

The aim of the study was to evaluate the anxiety levels of Polish and Turkish dentists during the COVID-19 pandemic and to investigate any relevant predictors. The main research objective was to investigate whether the COVID-19 pandemic correlated with the anxiety levels of dentists and to analyze the eventual differences between nationalities and the different conditions affecting the dentists during the pandemic.

### 1.3. Study Design

The investigation was an anonymous online questionnaire-based cross-sectional study conducted among dentists from two countries: Poland and Turkey. It was performed in accordance with the Public Opinion Research Guidelines and based on the computer-assisted web interview methodology. Ethical approval for this study was obtained.

## 2. Materials and Methods

### 2.1. Participants

The study was conducted among dentists: 200 in Poland and 200 in Turkey. Participation was voluntary and participants were allowed to terminate the survey at any time. Confidentiality and privacy were protected according to general data protection regulations. The study was conducted from 9 to 12 July 2021 and had a total of 400 respondents, 162 (40.5%) men and 238 (59.5%) women, aged between 23 and 67 years. The mean age of the dentists was 42.39 ± 9.99 years.

### 2.2. Data Collection

Online social media platforms, Facebook and Twitter, were employed for sampling. The survey was posted in the form of a link to a questionnaire that was to be filled in directly by a person willing to participate. The link was placed in professional social media groups (in which members were subject to prior verification confirming their dental licenses) and only to groups that allowed posting of this type of content.

### 2.3. Questionnaire

The survey consisted of two parts. Part 1 comprised demographic data, including age, gender, country of origin, COVID-19 infection history, place of work (private, public, mixed) and professional COVID-19 related lockdown information: governmental or other reasons (Appendix A, Table A1). Participants were asked if they had performed at least one COVID-19 test and if they had received at least one positive COVID-19 test result. They were also asked whether they had experienced any COVID-19 symptoms during the pandemic. Part 2 was based on the State–Trait Anxiety Inventory (STAI) [33,40]. STAI is a questionnaire-based psychological tool, which consists of 40 questions to be answered on a self-reporting basis. Answers are based on a 4-point Likert scale (1—not at all, 2—somewhat, 3—moderately, 4—very much). STAI measures two types of anxiety using subscales with 20 items each. State anxiety (anxiety about an event) assesses how the respondents felt during a stressful situation or a particular event and trait anxiety (anxiety as a personal characteristic) evaluates how they feel in general. The state anxiety subscale includes statements such as: “I am tense“, “I am worried”, “I feel calm”, “I feel secure”. There are phrases such as: “I worry too much over something that really doesn’t matter” and “I am a steady person” among the trait anxiety items. The range of scores for both of the subscales varies from 20 to 80, and a higher score indicates greater anxiety. A cut-off point of 39 has been suggested to detect clinically significant symptoms of anxiety and the comparison between subscale results enables the assessment of the difference between anxiety as a personal characteristic and anxiety caused by an event.

### 2.4. Statistical Methods

Continuous variables were summarized byt mean, standard deviation and frequency. Categorical variables were summarized by number and percentage of occurrences for each possible value. In order to obtain an effect size of 0.5 for determining the difference in numerical variables between two measurements, with the level of significance set to 0.05 and the power set to 0.9, the calculated minimum required sample size was 186 subjects per group. Normality was checked using the Shapiro–Wilk test. Yates’ correction was applied to prevent overestimation of statistical significance differences in numerical variables between the independent groups that were tested with a 95% confidence interval (CI). Significance was evaluated at the *p* < 0.05 level. The Student’s *t* test was used for the comparison of normally distributed parameters (quantitative data) between two groups, while the paired Student’s *t* test was applied for the comparison of the two types of anxiety. The Chi-square test was used to compare qualitative data. Correlation between two quantitative variables was assessed using the Pearson correlation coefficient (if both variables were normally distributed) and with the Spearman rank correlation coefficient (otherwise). Strength of relationship was interpreted as follows: |r| ≥ 0.9—very strong correlation, 0.7 ≤ |r| < 0.9—strong correlation, 0.5 ≤ |r| < 0.7—moderate correlation, 0.3 ≤ |r| < 0.5—weak correlation, |r| < 0.3—very week correlation, according to interpretation schema by Hinkle et al. [41]. Multivariate logistic regression was performed at the level *p* < 0.05, with a 95% confidence level (Cl) and cut-off point for clinically significant anxiety equal to 39. All the calculations were performed in IBM SPSS Statistics 22 (IBM SPSS, Turkey).

### 2.5. Ethics

The study was performed in accordance with the Helsinki Declaration and official approval from the Jagiellonian University Ethics Committee was obtained (No.1072.6120.158.2021).

## 3. Results

Cronbach’s alpha reliability factor for state anxiety on the STAI subscale was 0.86 in Poland and 0.84 in Turkey, while for trait anxiety on the STAI subscale it was 0.84 in both countries. The number of “survey invitation” social media post views and number of completed surveys within the study period were used to calculate the response rate, which was 71.5% for Poland and 74.3% for Turkey.

General evaluation of study parameters across both countries is presented in Table 1.

There was no statistically significant difference between Poland and Turkey in terms of gender (*p* > 0.05) and being tested for COVID-19 (*p* > 0.05). A total of 91.5% of positive COVID-19 test results among 59% of the tested dentists in Poland was found to be statistically significantly higher than in Turkey (24.1% of positive COVID-19 test results among 66.5% of tested respondents). The COVID-19 pandemic dental office lockdown count in Poland (0%) was found to be statistically significantly lower than in Turkey (59.5%). The dentists had to stop working temporary during the COVID-19 pandemic due to reasons other than lockdown (lack of protective supplies, COVID-19 infections, quarantine, etc.) more frequently in Poland (93%) than in Turkey (21%). In addition, the number of dentists who suffered from COVID-19 was found to be statistically significantly higher in Poland (54%) than in Turkey (16%). A total of 49.5% of infected dentists in Poland predicted that they were infected at work in comparison to 4.5% of Turkish dentists who suffered from COVID-19. On the contrary, 54% of dentists in Turkey stopped working due to the fear of infection, while in Poland only 13% of dentists were too afraid of contagion to decide to stop working.

Evaluation of state and trait anxiety levels across both countries is presented in Table 2.

Polish dentists presented mean trait and state anxiety levels that were statistically significantly lower than the Turkish ones (*p* = 0.001; *p* < 0.05); however, Polish dentists had a higher state anxiety level than trait anxiety level, while both types of anxiety among Turkish dentists were almost at the same level.

Evaluations of the state and trait anxiety scores using different factors separately for Poland and for Turkey are presented in Table 3 and Table 4. Spearman correlations are presented in Table A2 and Table A3.

Separate evaluation of the anxiety ranges for Poland and Turkey did not reveal statistically significant differences between the state and trait anxiety mean scores between dentists with and without a positive COVID-19 test result in Poland (*p* > 0.05) but such a difference existed among respondents in Turkey for the trait anxiety scores (*p* = 0.022). A similarity was observed in the correlation between both state and trait anxiety levels and the break at work caused by the COVID-19 pandemic. Anxiety level was found to be statistically significantly higher among dentists who had to stop working, than among those who did not have any breaks at work during the pandemic (39.65 and 43.27 vs. 31.64 and 34.21 in Poland; 48.74 and 49.38 vs. 46.65 and 46.99 in Turkey; *p* < 0.05). The results reported that a break at work caused by the fear of COVID-19 infection elicited a statistically significantly higher level of state and trait anxieties among Polish dentists (64.88; *p* = 0.001 and 68,96; *p* = 0.001), while this correlation among Turkish dentists was not statistically significant (*p* = 0.497 and *p* = 0.139).

Multivariate logistic regression of state and trait anxieties among Polish and Turkish dentists is presented in Table 5, Table 6, Table 7 and Table 8. Independent predictors of the odds of clinically significant state anxiety among Polish dentists are as follows (Table 5):

- Male sex (OR = 0.278): 72.2% decrease in chances compared to female sex,
- COVID-19 related breaks at work, other than governmental lockdowns (OR = 9.128): 9.128 times higher probability of anxiety,
- COVID-19 symptoms (OR = 0.409): 59.1% reduction in the odds,
- Private practice (OR = 0.264): 73.6% decrease in chances in comparison to a public one.

Independent predictors of the odds of clinically significant trait anxiety among Polish dentists are as follow (Table 6):

- Male sex (OR = 0.219): 73.6% decrease in chances in comparison to female sex,
- Private practice (OR = 0.405): 59.5% decrease in chances in comparison to a public one.

None of the variables analyzed by multivariate logistic regression were statistically significant independent predictors of the odds of clinically significant trait and state anxieties among Turkish dentists (Table 7 and Table 8).

## 4. Discussion

Our study reported that Polish dentists had higher state anxiety levels than trait anxiety levels, while both types of anxiety among Turkish dentists were almost at the same level. This might suggest that the state of the pandemic raised anxiety levels among Polish dentists more Turkish dentists. Several factors that were estimated in the study might explain the difference between the two countries. First of all, although a similar number of dentists in both countries were tested for COVID-19 (59% in Poland and 66.5% in Turkey), the number of positive test results was much higher in the Polish group (91.5% vs. 24.1%). In addition, the number of dentists who suffered from the COVID-19 symptoms was found to be higher in Poland (54%) than in Turkey (16%).

Evidence from previous pandemics showed that a lot of people were at risk of developing mental health disorders due to the COVID-19 pandemic, such as depression, anxiety, or sleep disturbances [13,14,15,16,17,18,19]. In a systemic review of the mental health status of patients during the COVID-19 pandemic the prevalence of depression was found to be 48%, which was higher than the pre-pandemic depression levels (5–34%) [25]. Additionally, the prevalence of anxiety and sleep disturbances among COVID-19 patients was reported to be higher than in the general population (31.9% and 20.1%, respectively) [42]. Recent studies have suggested that these disorders should be adequately investigated and addressed by clinicians in order to improve prognosis and avoid long-term mental health issues [5,6,7,8,9,10,11,12]. Therefore, there is a need for developing appropriate methods and tools for diagnosing mental health disorders associated with the pandemic. A recent study reported that anxiety especially should be carefully investigated and the work even suggested a new scale: the Coronavirus Anxiety Scale (CAS) [43].

Healthcare workers were one of the groups of people that were most affected by the pandemic; hence, they may present many different mental health problems as a consequence of it [44,45,46,47,48]. In one a study conducted in Turkey, the mean state anxiety score among emergency medical service professionals during the COVID-19 pandemic was 50.7 ± 11.6 [49], which was similar to our results (45.07 ± 10.94). As the state and trait anxiety scores may vary from 20 to 80, a cut-off point of 39–40 was suggested in order to detect clinically significant symptoms of anxiety [33,40]. In our study Polish dentists presented mean trait and state anxiety levels that were lower (39.09 ± 12.7, 42.64 ± 13.68) than the dentists in Turkey (47.09 ± 4.96, 47.49 ± 6.42), however both groups achieved levels over the cut-off point, which suggested clinically significant anxiety. Similar outcomes confirming the impact of the pandemic on the mental health of healthcare providers, and the higher stress and anxiety levels, has already been well documented in different studies during previous pandemics [13,14,15,16].

According to previous studies [17,36], we could expect a higher level of anxiety among infected individuals, thus the higher COVID-19 infection rate might be an explanation for the increased levels of state anxiety among Polish dentists versus the equal levels of state and trait anxieties in Turkey, where the infection rate was much lower. Secondly, the results revealed that as there was no governmental lockdown of dental practices in Poland (0%), compared with Turkey where the governmental lockdown affected 59.5% of dentists, the dentists in Poland worked longer in the pandemic conditions and experience more stress. Moreover, longer exposure to the contagious environment might have also been a factor in the higher infection rate among Polish dentists. Additionally many dentists in Poland (93%) had to stop working temporarily during the COVID-19 pandemic due to reasons than other lockdown, such as the lack of protective supplies, COVID-19 infection and quarantine, for example, which increased working stress factors. Multivariate logistic regression of the current study revealed that COVID-19-related breaks at work raised the possibility of clinically significant state anxiety among Polish dentists by a factor of 9.128. Finally, 49.5% of dentists in Poland who suffered from COVID-19 predicted they were infected at work in comparison to 4.5% of Turkish dentists. It has already been reported that the dental care environment carries the danger of contagion for dental professionals and, thus, the awareness of such risk may lead to stress and fear, especially in the pandemic circumstances [50]. Another study that demonstrated cross-sectional data of fear and anxiety among dental practitioners during the COVID-19 pandemic revealed that the high level of fear among dentists was explained by the high probability of close interaction with COVID-19 positive patients while they were treating them and by the awareness of the disease and its mortality [51].

Evaluating the anxiety ranges of our study separately for Poland and Turkey, we may observe less statistically significant correlations between some factors and anxiety in comparison to general analysis. There was no statistically significant differences between the state and trait anxiety mean scores between dentists with and without a positive COVID-19 test result in Poland (*p* > 0.05); however, such a difference existed among respondents in Turkey. State anxiety among Turkish dentists with positive COVID-19 test results was higher than those with a negative one. Moreover, Turkish dentists who suffered from a COVID-19 infection also presented a strong correlation and higher state and trait anxiety scores than Polish dentists who suffered from the disease. In the separate analysis for each country, a similarity was observed in the correlation between both state and trait anxiety levels and the break at work caused by the COVID-19 consequences. The anxiety level was found to be statistically significantly higher among dentists who had to stop working than among those who did not have any breaks at work during the pandemic. A recent study found that more than three-quarters of dental practitioners (78%) from 30 countries (including Poland and Turkey) were anxious and scared by the devastating effects of COVID-19 [50]. In our study 54% of dentists who took part in the survey in Turkey stopped working due to the fear of infection. Another study reported that 71.9% of questioned dental care providers were anxious about contagion and 85.4% of them revealed a fear of infecting others. Moreover, respondents, who were constantly working during the pandemic, felt significantly more stressed about the possibility of infection while reporting that their workplaces handled the outbreak well (80%) [52]. The results of our study demonstrated that a break at work caused by the fear of COVID-19 infection elicited a statistically significant higher level of state and trait anxieties among Polish dentists.

Many recent studies have reported similar findings regarding the psychological consequences of the COVID-19 pandemic for dental health professionals. The prevalence of anxiety among frontline dental staff was reported to be 46.4% in a study by Chen et al. [30]. In another study 42.5% of dentists had COVID-19-associated anxiety and 35% of them had general health disorders [28]. Gasparro et al. [27] indicated that job insecurity and fear of COVID-19 were positively associated with depressive symptoms among dentists. In an online questionnaire-based survey assessing the level of perceived stress among dentists (before the COVID-19 pandemic and immediately after the nationwide lockdown was announced) the increase was noticed: the level of perceived stress rose from 18.61 to 20.72 during the outbreak (PSS-perceived stress scale) [29]. Before the pandemic having no family time due to long working hours (90%) was the major stressor among dentists, while concerns about getting infected (83.3%), followed by stress over financial implications were identified as the most frequent stressors during the outbreak. In the review by Sharma et al. [26] the psychological consequences were discussed in detail to highlight the challenges dentists were facing during the pandemic. According to the authors, compromised mental health was an important area that requirds attention during the COVID-19 outbreak.

The obtained results revealed that Polish and Turkish dentists experienced mental health issues during the COVID-19 pandemic with slight differences among the evaluated factors; however, regardless the factors and the way the data was analyzed, the anxiety levels remained high and over the cut-off point in most cases. A safe working environment and adequate infection control were important factors in securing less fear and anxiety. The long-term high level of anxiety among dentists may lead not only to significant economic implications but also to mental health disorders. Thus, in the situation of fear and anxiety caused by the pandemic, it was important to provide the adequate psychological coping mechanisms and strategies for diagnosing and maintaining mental health. Measuring anxiety levels and analyzing the related factors seemed to be useful tools in diagnosing and coping with the mental health disorders during pandemic.

The study has several limitations. First of all, it was carried out in two countries and, due to the COVID-19 pandemic, in an online form. This, and also the small number of respondents, may have limited t the generalization of the results. Furthermore, the as State and Trait Anxiety Inventory consists of 40 questions, in order to keep the survey relevant and not too overwhelming, only a few selected influencing factors out of many were included in the study. Additionally, our research was conducted in a short period of time and, thus, it did not examine the influence of the dynamics and duration of the pandemic on measured parameters. Additionally STAI assessment relies only on self-perceived data, which have a strong subjective component in the way answers have been resolved by the patient. As a non-probabilistic sampling method was used (the survey was posted as an open invitation on social media within a verified group of professionals) the calculation of a response rate, as well as the number of potential readers, was based on the number of post reads and completed surveys within the study period. However, as we did not have any basic information about the dentists who read the post but did not take part in the survey, the selection bias may have had an influence on the study estimates. Finally, although the authors identified the survey goals and clearly defined requirements for the target audience to the best of their ability, it was very difficult to estimate how representative the study participants were for all dentists in Poland and Turkey. Further longitudinal studies on a larger group of respondents, and in many countries, would definitely provide more detailed data.

## 5. Conclusions

The present study reported that Polish and Turkish dentists were suffering from anxiety during the COVID-19 pandemic. The study revealed that dentists in Poland were more mentally affected by the COVID-19 pandemic than Turkish ones, which might have been caused by a higher coronavirus infection rate. Lack of governmental lockdowns of dental practices during the outbreak in Poland, contrary to Turkey, may also have explained the higher level of anxiety due to the long-term exposure to hard working conditions. The stress at work seemed to be greater for Polish dentists as many of them had faced a temporary practice closure due to other pandemic related factors, such as the lack of protective supplies, COVID-19 infection and quarantine. Finally, the research confirmed previous reports of a high rate of fear of being infected at work. More studies could be effective in analyzing factors influencing anxiety among dentists during the pandemic.

## Figures and Tables

**Table 1:** Evaluation of study parameters in Turkey and Poland.

		Country of Residence		Total	*p*
		Poland	Turkey		
		Mean ± SD	Mean ± SD	Mean ± SD	
Age:		41.32 ± 8.88	43.46 ± 10.91	42.39 ± 9.99	^1^ t = 2.152 d = 0.215 *p* = 0.032 *
		*n* (%)	*n* (%)	*n* (%)	
Gender	Male	74 (37%)	88 (44%)	162 (40.5%)	^2^ chi2 = 2.033 *p* = 0.154
	Female	126 (63%)	112 (56%)	238 (59.5%)	
COVID-19 test	Yes	118 (59%)	133 (66.5%)	251 (62.7%)	^2^ chi2 = 2.406 *p* = 0.121
	No	82 (41%)	67 (33.5%)	149 (37.3%)	
Positive COVID-19 test result (*n* = 251)	Yes	108 (91.5%)	32 (24.1%)	140 (55.8%)	^3^ chi2 = 63.473 *p* = 0.001 *
	No	10 (8.5%)	101 (75.9%)	111 (44.2%)	
Professional governmental COVID-19 lockdown	Yes	0 (0%)	119 (59.5%)	119 (29.8%)	^3^ chi2 = 169.395 *p* = 0.001 *
	No	200 (100%)	81 (40.5%)	281 (70.3%)	
Other COVID-19 related breaks at work	Yes	186 (93%)	42 (21%)	228 (57%)	^3^ chi2 = 211.506 *p* = 0.001 *
	No	14 (7%)	158 (79%)	172 (43%)	
COVID-19 disease	Yes	108 (54%)	32 (16%)	140 (35%)	^2^ chi2 = 63.473 *p* = 0.001 *
	No	92 (46%)	168 (84%)	260 (65%)	
Possible infection at work	Yes	99 (49.5%)	9 (4.5%)	108 (27%)	^2^ chi2 = 178.240 *p* = 0.001 *
	No	0 (0%)	86 (43%)	86 (21.5%)	
	^4^ DN	9 (4.5%)	34 (17%)	43 (10.8%)	
	^5^ NA	92 (46%)	71 (35.5%)	163 (40.8%)	
Not working due to the fear of COVID-19	Yes	26 (13%)	108 (54%)	134 (33.5%)	^2^ chi2 = 75.457 *p* = 0.001 *
	No	174 (87%)	92 (46%)	266 (66.5%)	
Place of work	Private	83 (41.5%)	127 (63.5%)	210 (52.5%)	^2^ chi2 = 20.633 *p* = 0.001 *
	Public	51 (25.5%)	38 (19%)	89 (22.3%)	
	Mixed	66 (33%)	35 (17.5%)	101 (25.3%)	

^1^ Student’s *t* test; ^2^ Chi-square test; ^3^ continuity (Yates) correction; ^4^ DN—I do not know; ^5^ NA—not applicable; * *p* < 0.05.

**Table 2:** Evaluation of state and trait anxiety levels in Turkey and Poland (* *p* < 0.05).

	Country of Residence		Total	*p* (Student *t* Test)
	Poland	Turkey		
	Mean ± SD	Mean ± SD	Mean ± SD	
Trait Anxiety	39.09 ± 12.7	47.09 ± 4.96	43.09 ± 10.43	t = 8.301 d = 0.830 *p* = 0.001 *
State Anxiety	42.64 ± 13.68	47.49 ± 6.42	45.07 ± 10.94	t = 4.539 d = 0.454 *p* = 0.001 *
*p* (paired Student *t* Test)	t = 16.395 d = 1.159 *p* < 0.001 *	t = 0.989 d = 0.070 *p* = 0.15		

**Table 3:** Evaluation of state and trait anxiety levels using the study parameters for Poland.

			Trait Anxiety	State Anxiety
Poland		*n*	Mean ± SD	Mean ± SD
Gender	F	126	41.41 ± 12.98	45.57 ± 13.07
	M	74	35.12 ± 11.19	37.64 ± 12.15
	*p*		t = 2.224	t = 2.460
			d = 0.446	d = 0.427
			0.032 *	0.032 *
Positive COVID-19 test result	Yes	108	36.6 ± 8.06	40.68 ± 9.81
	No	10	36.4 ± 17.23	39.8 ± 17.9
	*p*		t = 0.067 d = 0.022 *p* = 0.972	t = 0.249 d = 0.082 *p* = 0.882
Professional governmental COVID-19 lockdown	Yes	0	-	-
	No	200	39.09 ± 12.7	42.64 ± 13.68
	*p*		-	-
Other COVID-19 related breaks at work	Yes	186	39.65 ± 12.86	43.27 ± 13.82
	No	14	31.64 ± 7.09	34.21 ± 7.95
	*p*		t = 2.299 d = 0.637 *p* = 0.003 *	t = 2.419 d = 0.670 *p* = 0.001 *
COVID-19 disease	Yes	108	36.6 ± 8.06	40.68 ± 9.81
	No	92	42.0 ± 16.13	44.95 ± 16.92
	*p*		t = 3.059 d = 0.434 *p* = 0.004 *	t = 2.222 d = 0.315 *p* = 0.035 *
Not working due to the fear of COVID-19	Yes	26	64.88 ± 8.39	68.96 ± 7.52
	No	174	35.23 ± 7.76	38.71 ± 9.35
	*p*		t = 17.983 d = 3.781 *p* = 0.001 *	t = 15.746 d = 3.311 *p* = 0.001 *

Student’s *t* Test, * *p* < 0.05.

**Table 4:** Evaluation of state and trait anxiety levels using the study parameters for Turkey.

			Trait Anxiety	State Anxiety
Turkey		*n*	Mean ± SD	Mean ± SD
Gender	F	112	46.25 ± 4.81	47.80 ± 5.53
	M	88	48.14 ± 4.98	47.09 ± 7.41
	*p*		t = 1.425	t = 1.335
			d = 0.275	d = 0.192
			0.132	0.132
Positive COVID-19 test result	Yes	32	48.94 ± 6.1	48.97 ± 6.98
	No	101	46.64 ± 4.44	46.6 ± 6.8
	*p*		t = 2.224 d = 0.446 *p* = 0.022 *	t = 1.825 d = 0.305 *p* = 0.091
Professional governmental COVID-19 lockdown	Yes	119	47.47 ± 5.13	48.08 ± 7.43
	No	81	46.52 ± 4.67	46.62 ± 4.44
	*p*		t = 1.335 d = 0.192 *p* = 0.183	t = 1.592 d = 0.229 *p* = 0.083
Other COVID-19 related breaks at work	Yes	42	48.74 ± 5.14	49.38 ± 5.49
	No	158	46.65 ± 4.83	46.99 ± 6.57
	*p*		t = 2.460 d = 0.427 *p* = 0.015 *	t = 2.167 d = 0.376 *p* = 0.031 *
COVID-19 disease	Yes	32	48.94 ± 6.1	48.97 ± 6.98
	No	168	46.73 ± 4.65	47.21 ± 6.29
	*p*		t = 2.330 d = 0.449 *p* = 0.021 *	t = 1.425 d = 0.275 *p* = 0.156
Not working due to the fear of COVID-19	Yes	108	47.31 ± 5.1	48.11 ± 5.27
	No	92	46.83 ± 4.81	46.76 ± 7.52
	*p*		t = 0.680 d = 0.097 *p* = 0.497	t = 1.487 d = 0.211 *p* = 0.139

Student’s *t* Test, * *p* < 0.05.

**Table 5:** Multivariate logistic regression of state anxiety among Polish dentists.

Variable		OR	95% CI		*p*
Age	[years]	0.996	0.962	1.032	0.841
Gender	Female	1	ref.		
	Male	0.278	0.135	0.574	0.001 *
Other COVID-19 related breaks at work	No	1	ref.		
	Yes	9.128	1.664	50.076	0.011 *
COVID-19 symptoms	No	1	ref.		
	Yes	0.409	0.203	0.824	0.012 *
Place of work	Public	1	ref.		
	Private	0.264	0.117	0.593	0.001 *
	Mixed	0.561	0.247	1.27	0.165

*p*—multivariate logistic regression; * level of statistical significance *p* < 0.05.

**Table 6:** Multivariate logistic regression of trait anxiety among Polish dentists.

Variable		OR	95% CI		*p*
Age	[years]	1.002	0.968	1.037	0.912
Gender	Female	1	ref.		
	Male	0.219	0.111	0.432	<0.001 *
Other COVID-19 related breaks at work	No	1	ref.		
	Yes	3.916	0.969	15.833	0.055
COVID-19 disease	No	1	ref.		
	Yes	0.591	0.296	1.181	0.137
Place of work	Public	1	ref.		
	Private	0.405	0.184	0.891	0.025 *
	Mixed	0.966	0.422	2.209	0.934

*p*—multivariate logistic regression; * level of statistical significance *p* < 0.05.

**Table 7:** Multivariate logistic regression of state anxiety among Turkish dentists.

Variable		OR	95% CI		*p*
Age	[years]	1.021	0.97	1.075	0.423
Gender	Female	1	ref.		
	Male	1.712	0.519	5.65	0.378
Professional govermental COVID-19 lockdown	No	1	ref.		
	Yes	0.838	0.271	2.591	0.758
Other COVID-19 related breaks at work	No	1	ref.		
	Yes	1.794	0.372	8.657	0.467
COVID-19 symptoms	No	1	ref.		
	Yes	1.281	0.263	6.248	0.759
Place of work	Public	1	ref.		
	Private	0.224	0.028	1.807	0.16
	Mixed	0.572	0.047	6.941	0.661

*p*—multivariate logistic regression.

**Table 8:** Multivariate logistic regression of trait anxiety among Turkish dentists.

Variable		OR	95% CI		*p*
Age	[years]	0.97	0.901	1.044	0.421
Gender	Female	1	ref.		
	Male	1.294	0.3	5.575	0.729
Professional govermental COVID-19 lockdown	No	1	ref.		
	Yes	0.533	0.125	2.274	0.395
Other COVID-19 related breaks at work	No	1	ref.		
	Yes	20797313.175	0	Inf	0.992
COVID-19 disease	No	1	ref.		
	Yes	0.701	0.128	3.841	0.682
Place of work	Public	1	ref.		
	Private	0.534	0.06	4.764	0.575
	Mixed	0.845	0.048	14.842	0.908

*p*—multivariate logistic regression.

## Data Availability

The data presented in this study are available on request from the corresponding author. The data are not publicly available due to ethical restrictions.

## Appendix A

**Table A1:** Part 1 of the study survey.

Age [years]	
Country of origin	
Gender	Male
	Female
Have you been tested for the COVID-19 at least once?	Yes
	No
Did you have any positive COVID-19 test results?	Yes
	No
Did you suffer any professional governmental lockdowns during the COVID-19 pandemic?	Yes
	No
Did you have to stop working due to reasons other than governmental lockdowns that were COVID-19 related (lack of protective supplies, COVID-19 infections yours or your co-workers quarantine, etc.)?	Yes
	No
Did you suffer from COVID-19 symptoms?	Yes
	No
If so, do you think you were infected at work?	Yes
	No
	I do not know
	Not applicable
Did you stop working during the COVID-19 pandemic due to the fear of getting infected?	Yes
	No
Place of work	Private
	Public
	Mixed

**Table A2:** COVID-19 related factors and state and trait anxiety correlations for Poland.

COVID-19 Factor			State and Trait Anxiety Correlation			
	Trait Anxiety Correlation Coefficient	*p*	Strength	State Anxiety Correlation Coefficient	*p*	Strength
Positive COVID-19 test result	0.256	0.972	-	0.016	0.882	-
Professional governmental COVID-19 lockdown	0.289	-	-	0.001	-	-
Other COVID-19 related breaks at work	0.776	0.023	Strong correlation	0.705	0.001	Strong correlation
COVID-19 disease	0.378	0.004	Weak correlation	0.309	0.035	Weak correlation
Not working due to the fear of COVID-19	0.731	0.001	Strong correlation	0.708	0.001	Strong correlation

**Table A3:** COVID-19 related factors and state and trait anxiety correlations for Turkey.

COVID-19 Factor			State and Trait Anxiety Correlation			
	Trait Anxiety Correlation Coefficient	*p*	Strength	State Anxiety Correlation Coefficient	*p*	Strength
Positive COVID-19 test result	0.356	0.022	Weak correlation	0.389	0.091	-
Professional governmental COVID-19 lockdown	0.589	0.183	-	0.578	0.083	-
Other COVID-19 related breaks at work	0.576	0.015	Moderate correlation	0.508	0.031	Moderate correlation
COVID-19 disease	0.578	0.021	Moderate correlation	0.492	0.156	-
Not working due to the fear of COVID-19	0.597	0.497	-	0.467	0.139	-

## References

[1] WHO (2020). WHO Announces COVID-19 Outbreak a Pandemic. http://www.euro.who.int/en/health-topics/healthemergencies/coronavirus-covid-19/news/news/2020/3/whoannounces-covid-19-outbreak-a-pandemic.

[2] Khajuria A., Tomaszewski W., Liu Z., Chen J., Mehdian R., Fleming S., Vig S., Crawford M.J. (2021). Workplace Factors Associated with Mental Health of Healthcare Workers during the COVID-19 Pandemic: An International Cross-Sectional Study. BMC Health Serv. Res..

[3] Yao H., Chen J.-H., Xu Y.-F. (2020). Patients with Mental Health Disorders in the COVID-19 Epidemic. Lancet Psychiatr..

[4] Xiang Y.-T., Yang Y., Li W., Zhang L., Zhang Q., Cheung T., Ng C.H. (2020). Timely Mental Health Care for the 2019 Novel Coronavirus Outbreak Is Urgently Needed. Lancet Psychiatr..

[5] Shah S.M.A., Mohammad D., Qureshi M.F.H., Abbas M.Z., Aleem S. (2020). Prevalence, Psychological Responses and Associated Correlates of Depression, Anxiety and Stress in a Global Population, During the Coronavirus Disease (COVID-19) Pandemic. Commun. Ment. Health J..

[6] Bäuerle A., Teufel M., Musche V., Weismüller B., Kohler H., Hetkamp M., Dörrie N., Schweda A., Skoda E.-M. (2020). Increased Generalized Anxiety, Depression and Distress during the COVID-19 Pandemic: A Cross-Sectional Study in Germany. J. Public Health.

[7] Rehman U., Shahnawaz M.G., Khan N.H., Kharshiing K.D., Khursheed M., Gupta K., Kashyap D., Uniyal R. (2020). Depression, Anxiety and Stress Among Indians in Times of Covid-19 Lockdown. Commun. Ment. Health J..

[8] Shi J., Gao Y., Zhao L., Li Y., Yan M., Niu M.M., Chen Y., Song Z., Zhang R., Zhang L. (2020). Prevalence of Delirium, Depression, Anxiety, and Post-Traumatic Stress Disorder among COVID-19 Patients: Protocol for a Living Systematic Review. Syst. Rev..

[9] Zhang J., Yang Z., Wang X., Li J., Dong L., Wang F., Li Y., Wei R., Zhang J. (2020). The Relationship between Resilience, Anxiety and Depression among Patients with Mild Symptoms of COVID-19 in China: A Cross-Sectional Study. J. Clin. Nurs..

[10] Kibbey M.M., Fedorenko E.J., Farris S.G. (2021). Anxiety, Depression, and Health Anxiety in Undergraduate Students Living in Initial US Outbreak “Hotspot” during COVID-19 Pandemic. Cogn. Behav. Ther..

[11] Akgor U., Fadıloglu E., Soyak B., Unal C., Cagan M., Temiz B.E., Erzenoglu B.E., Ak S., Gultekin M., Ozyuncu O. (2021). Anxiety, Depression and Concerns of Pregnant Women during the COVID-19 Pandemic. Arch. Gynecol. Obstet..

[12] Kujawa A., Green H., Compas B.E., Dickey L., Pegg S. (2020). Exposure to COVID-19 Pandemic Stress: Associations with Depression and Anxiety in Emerging Adults in the United States. Depress. Anxiety.

[13] Maunder R., Hunter J., Vincent L., Bennett J., Peladeau N., Leszcz M., Sadavoy J., Verhaeghe L.M., Steinberg R., Mazzulli T. (2003). The Immediate Psychological and Occupational Impact of the 2003 SARS Outbreak in a Teaching Hospital. Can. Med. Assoc. J..

[14] Lee A.M., Wong J.G., McAlonan G.M., Cheung V., Cheung C., Sham P.C., Chu C.-M., Wong P.-C., Tsang K.W., Chua S.E. (2007). Stress and Psychological Distress among SARS Survivors 1 Year after the Outbreak. Can. J. Psychiatr..

[15] Chua E.S., Cheung V., Cheung C., McAlonan G.M., Wong J.W., Cheung E.P., Chan M.T., Wong M.M., Tang S.W., Choy K.M. (2004). Psychological Effects of the SARS Outbreak in Hong Kong on High-Risk Health Care Workers. Can. J. Psychiatr..

[16] Chong M.-Y., Wang W.-C., Hsieh W.-C., Lee C.-Y., Chiu N.-M., Yeh W.-C., Huang O.-L., Wen J.-K., Chen C.-L. (2004). Psychological Impact of Severe Acute Respiratory Syndrome on Health Workers in a Tertiary Hospital. Br. J. Psychiatr..

[17] Koh D., Lim M.K., Chia S.E., Ko S.M., Qian F., Ng V., Tan B.H., Wong K.S., Chew W.M., Tang H.K. (2005). Risk Perception and Impact of Severe Acute Respiratory Syndrome (SARS) on Work and Personal Lives of Healthcare Workers in Singapore: What Can We Learn?. Med. Care.

[18] Wu P., Fang Y., Guan Z., Fan B., Kong J., Yao Z., Liu X., Fuller C.J., Susser E., Lu J. (2009). The Psychological Impact of the SARS Epidemic on Hospital Employees in China: Exposure, Risk Perception, and Altruistic Acceptance of Risk. Can. J. Psychiatr..

[19] Maunder R.G., Lancee W.J., Balderson K.E., Bennett J.P., Borgundvaag B., Evans S., Fernandes C.M.B., Goldbloom D.S., Gupta M., Hunter J.J. (2006). Long-Term Psychological and Occupational Effects of Providing Hospital Healthcare during SARS Outbreak. Emerg. Infect. Dis..

[20] Sampaio-Maia B., Caldas I., Pereira M., Pérez-Mongiovi D., Araujo R. (2016). The Oral Microbiome in Health and Its Implication in Oral and Systemic Diseases. Adv. Appl. Microbiol..

[21] Yamashita Y., Takeshita T. (2017). The Oral Microbiome and Human Health. J. Oral Sci..

[22] Gao L., Xu T., Huang G., Jiang S., Gu Y., Chen F. (2018). Oral Microbiomes: More and more Importance in Oral Cavity and Whole Body. Protein Cell.

[23] Jia G., Zhi A., Lai P.F.H., Wang G., Xia Y., Xiong Z.-Q., Zhang H., Che N., Ai L. (2018). The Oral Microbiota–A Mechanistic Role for Systemic Diseases. Br. Dent. J..

[24] Gomez A., Nelson K.E. (2016). The Oral Microbiome of Children: Development, Disease, and Implications beyond Oral Health. Microb. Ecol..

[25] Vergara-Buenaventura A., Chavez-Tuñon M., Castro-Ruiz C. (2020). The Mental Health Consequences of Coronavirus Disease 2019 Pandemic in Dentistry. Disaster Med. Public Health Prep..

[26] Sharma S., Parolia A., Kanagasingam S. (2020). A Review on COVID-19 Mediated Impacts and Risk Mitigation Strategies for Dental Health Professionals. Eur. J. Dent..

[27] Gasparro R., Scandurra C., Maldonato N.M., Dolce P., Bochicchio V., Valletta A., Sammartino G., Sammartino P., Mariniello M., Di Lauro A.E. (2020). Perceived Job Insecurity and Depressive Symptoms among Italian Dentists: The Moderating Role of Fear of COVID-19. Int. J. Environ. Res. Public Health.

[28] Salehiniya H., Abbaszadeh H. (2021). Prevalence of Corona-Associated Anxiety and Mental Health Disorder among Dentists during the COVID-19 Pandemic. Neuropsychopharmacol. Rep..

[29] Mishra S., Singh S., Tiwari V., Vanza B., Khare N., Bharadwaj P. (2020). Assessment of Level of Perceived Stress and Sources of Stress among Dental Professionals Before and during the COVID-19 Outbreak. J. Int. Soc. Prev. Commun. Dent..

[30] Chen Y., Li W. (2021). Influencing Factors Associated with Mental Health Outcomes Among Dental Medical Staff in Emergency Exposed to Coronavirus Disease 2019: A Multicenter Cross-Sectional Study in China. Front. Psychiatr..

[31] Collin V., O’selmo E., Whitehead P. (2021). Psychological Distress and the Perceived Impact of the COVID-19 Pandemic on UK Dentists during a National Lockdown. Br. Dent. J..

[32] Tao J., Lin Y., Jiang L., Zhou Z., Zhao J., Qu D., Li W., Zhu Y. (2020). Psychological Impact of the COVID-19 Pandemic on Emergency Dental Care Providers on the Front Lines in China. Int. Dent. J..

[33] Hacimusalar Y., Kahve A.C., Yasar A.B., Aydin M.S. (2020). Anxiety and Hopelessness Levels in COVID-19 Pandemic: A Comparative Study of Healthcare Professionals and other Community Sample in Turkey. J. Psychiatr. Res..

[34] González-Olmo M.J., Ortega-Martínez A.R., Delgado-Ramos B., Romero-Maroto M., Carrillo-Diaz M. (2020). Perceived Vulnerability to Coronavirus Infection: Impact on Dental Practice. Braz. Oral Res..

[35] (2020). United Nations Human Development Report.

[36] Polish Medical Chamber (2020). Statistics.

[37] Turkish Dental Association (2020). The Organization: Ankara, Turkey. http://tdb.org.tr/sag_menu_goster.php?Id=455.

[38] Ministry of Health (2021). Coronavirus Report.

[39] Turkish Ministry of Health (2021). Coronavirus Tables.

[40] Wrześniewski K., Sosnowski T., Jaworowska A., Fecenec D. (2011). STAI–Inwentarz Stanu i Cechy Lęku. Polska Adaptacja.

[41] Hinkle D.E., Wiersma W., Jurs S.G. (2003). Applied Statistics for the Behavioral Sciences.

[42] Deng J., Zhou F., Hou W., Silver Z., Wong C.Y., Chang O., Huang E., Zuo Q.K. (2020). The Prevalence of Depression, Anxiety, and Sleep Disturbances in COVID-19 Patients: A Meta-Analysis. Ann. N. Y. Acad. Sci..

[43] Lee S.A. (2020). Coronavirus Anxiety Scale: A Brief Mental Health Screener for COVID-19 Related Anxiety. Death Stud..

[44] Danet A.D. (2021). Impacto Psicológico de la COVID-19 en Profesionales Sanitarios de Primera Línea en el Ambito Occidental. Una Revisión Sistemática..

[45] Thatrimontrichai A., Weber D.J., Apisarnthanarak A. (2021). Mental Health among Healthcare Personnel during COVID-19 in Asia: A Systematic Review. J. Formos. Med. Assoc..

[46] Lai J., Ma S., Wang Y., Cai Z., Hu J., Wei N., Wu J., Du H., Chen T., Li R. (2020). Factors Associated with Mental Health Outcomes among Health Care Workers Exposed to Coronavirus Disease 2019. JAMA Netw. Open.

[47] Cai C.Z., Lin Y.-L., Hu Z.-J., Wong L.P. (2021). Psychological and Mental Health Impacts of COVID-19 Pandemic on Healthcare Workers in China: A Review. World J. Psychiatr..

[48] Smallwood N., Willis K. (2021). Mental Health among Healthcare Workers during the COVID-19 Pandemic. Respirology.

[49] Usul E., Şan I., Bekgöz B. (2020). The Effect of the COVID-19 Pandemic on the Anxiety Level of Emergency Medical Services Professionals. Psychiatr. Danub..

[50] Ahmed M.A., Jouhar R., Ahmed N., Adnan S., Aftab M., Zafar M.S., Khurshid Z. (2020). Fear and Practice Modifications among Dentists to Combat Novel Coronavirus Disease (COVID-19) Outbreak. Int. J. Environ. Res. Public Health.

[51] Suryakumari V., Reddy Y.P., Yadav S.S., Doshi D., Reddy V.S. (2020). Assessing Fear and Anxiety of Corona Virus among Dental Practitioners. Disaster Med. Public Health Prep..

[52] Uhlen M.M., Ansteinsson V.E., Stangvaltaite-Mouhat L., Korzeniewska L., Skudutyte-Rysstad R., Shabestari M., Mdala I., Hovden E.A.S. (2021). Psychological Impact of the COVID-19 Pandemic on Dental Health Personnel in Norway. BMC Health Serv. Res..

